# Anaplerotic Role of Glucose in the Oxidation of Endogenous Fatty Acids during Dengue Virus Infection

**DOI:** 10.1128/mSphere.00458-17

**Published:** 2018-01-31

**Authors:** Lorena O. Fernandes-Siqueira, Julianna D. Zeidler, Bruna G. Sousa, Thiago Ferreira, Andrea T. Da Poian

**Affiliations:** a Laboratory of Viral Biochemistry, Instituto de Bioquímica Médica Leopoldo de Meis, Universidade Federal do Rio de Janeiro, Rio de Janeiro, Brazil; Icahn School of Medicine at Mount Sinai

**Keywords:** Crabtree effect, dengue virus, energy metabolism, fatty acid oxidation, high-resolution respirometry, mitochondrial function

## Abstract

Dengue virus (DENV) is among the most important human arboviruses and is clinically and experimentally associated with lipid metabolism disorders. Using high-resolution respirometry, we analyzed the metabolic switches induced by DENV in a human hepatic cell line. This experimental approach allowed us to determine the contribution of fatty acids, glutamine, glucose, and pyruvate to mitochondrial bioenergetics, shedding light on the mechanisms involved in DENV-induced metabolic alterations. We found that while infection strongly inhibits glutamine oxidation, it increases the cellular capacity of metabolizing glucose; remarkably, though, this substrate, instead being used as an energy source, performs an anaplerotic role in the oxidation of endogenous lipids. Fatty acids become the main energetic substrate in infected cell, and through the pharmacological modulation of β-oxidation we demonstrated that this pathway is essential for virus replication. Interestingly, infected cells were much less susceptible to the Crabtree effect, i.e., the glucose-mediated inhibition of mitochondrial oxygen consumption, suggesting that infection favors cellular respiration by increasing ADP availability.

**IMPORTANCE** Dengue virus infection is a major cause of human arbovirosis, for which clinical and experimental evidence supports the idea that liver dysfunction and lipid metabolism disorders are characteristics of severe disease. Analyzing mitochondrial bioenergetics, here we show that infection of hepatic cells with dengue virus favors the cellular capacity of metabolizing glucose, impairing the normal metabolic flexibility that allows the oxidative machinery to switch among the main energetic substrates. However, instead of being used as an energy source, glucose performs an anaplerotic role in the oxidation of endogenous fatty acids, which become the main energetic substrate during infection. Taken together, the results shed light on metabolic mechanisms that may explain the profound alterations in lipid metabolism for severe dengue patients, contributing to the understanding of dengue physiopathology.

## Abstract (precis)

Dengue virus infection is a major cause of human arbovirosis, for which clinical and experimental evidence supports the idea that liver dysfunction and lipid metabolism disorders are characteristics of severe disease. Analyzing mitochondrial bioenergetics, here we show that infection of hepatic cells with dengue virus favors the cellular capacity of metabolizing glucose, impairing the normal metabolic flexibility that allows the oxidative machinery to switch among the main energetic substrates. However, instead of being used as an energy source, glucose performs an anaplerotic role in the oxidation of endogenous fatty acids, which become the main energetic substrate during infection. Taken together, the results shed light on metabolic mechanisms that may explain the profound alterations in lipid metabolism for severe dengue patients, contributing to the understanding of dengue physiopathology.

## INTRODUCTION

Since viruses completely depend on host metabolic pathways to yield energy and biosynthetic precursors for their replication, reprogramming cellular metabolism is a strategy used by many viruses to ensure a successful spread in the infected organism. About 50 years ago, the studies on virus-induced metabolic alterations were mainly focused on the glycolytic metabolism in cells infected by transforming viruses, such as murine sarcoma or Rous sarcoma viruses (1, 2). Most of the articles reported that these viruses stimulated the glucose uptake and glycolytic flux in the infected cells, an effect similar to that observed for cancer cells, as described by Otto Warburg at the beginning of the 20th century (3). More recently, reports of metabolic reprogramming induced by virus infection reemerged in the scientific literature, the broadening the range of viral families and metabolic aspects studied (4), including changes in mitochondrial bioenergetics (5) and in lipid metabolism (6, 7).

Dengue virus (DENV) infections are endemic in more than 100 countries, with recent estimates predicting about 400 million infections occurring each year and more than 3 billion people living in risk areas (8). DENV, a member of the *Flaviviridae* family, is an enveloped virus with a positive-sense single-stranded genomic RNA that encodes three structural proteins (capsid [C], membrane [prM], and envelope [E]) and seven nonstructural proteins (NS1, NS2A, NS2B, NS3, NS4A, NS4B, and NS5). The diseases caused by DENV range from a mild fever to life-threatening severe diseases, known as dengue hemorrhagic fever (DHF) and dengue shock syndrome (DSS), which are characterized by an increase in vascular endothelial permeability that leads to plasma leakage and may evolve into a fatal hypovolemic shock (9).

In recent years, an increasing number of clinical and *in vitro* studies by our group and other groups reported DENV-induced metabolic alterations. Clinical evidence indicates that lipid metabolism is deeply affected in dengue patients, especially in severe cases of infection. Analyses of sera in different populations in India, Singapore, Nicaragua, Venezuela, and Brazil revealed an increase in the serum levels of free fatty acids and acyl-carnitine (10) and a reduction in the levels of circulating lipoproteins in patients afflicted by the most severe forms of dengue (11–14). Additionally, necropsy samples from dengue patients (15–17) as well as animal models for DENV infection (18, 19) showed macrovesicular and microvesicular hepatic steatosis. Changes in serum amino acid concentrations, in particular, decreases in glutamine and histidine concentrations and increases in tyrosine and phenylalanine concentrations, were also observed (10, 14).

*In vitro* studies using different types of cells showed alterations in distinct metabolic pathways during DENV infection, such as modulation of glycolysis (20), mitochondrial dysfunction (21), activation of fatty acid synthesis (22), lipid droplet (LD) accumulation (23, 24), and increased autophagy-mediated mobilization of fatty acids (6). One of the consequences of these virus-induced alterations of the metabolic pathways is that the infected cells may change the preference for oxidizing specific energetic substrates despite the nutrient availability, which may ultimately result in the impairment of cellular functions and/or in damage to the organism. In this context, exploring mitochondrial function is an interesting approach for understanding the mechanisms behind the observed metabolic alterations that occur during infection.

Although some ATP is synthesized anaerobically by glycolysis in the cytoplasm even in the presence of oxygen, most cellular ATP is produced by oxidative phosphorylation, a process mediated by membrane-bound protein complexes located in the inner mitochondrial membrane, which form the electron transport system (ETS). The oxidation of different substrates generates NADH and reduced flavin adenine dinucleotide (FADH_2_), which transfer the electrons to oxygen through the ETS. Thus, measuring the oxygen consumption by cells under a condition of interest (for instance, after incubation with a specific substrate) allows one to investigate the cellular metabolic status. Using this approach, our group has demonstrated that the infection of a human hepatic cell with DENV results in increased respiration uncoupled to ATP synthesis, which could be correlated to apoptosis induction (21). However, the metabolic switch occurring among the different substrates as well as its correlation with the overall metabolic alterations observed during infection remained to be investigated. It is important to have in mind that one of the main limitations with respect to deep understanding of virus-induced metabolic switches *in vitro* is the influence of the nutrients already present in the culture medium, which in fact represents an artificial situation. One example is the Crabtree effect, a complex phenomenon that results in the inhibition of cellular respiration in the presence of glucose (25), since most *in vitro* experiments are carried out in media containing glucose.

Here, we used high-resolution respirometry to analyze the metabolic switches that occur during infection of Huh7, a human hepatic cell lineage, by DENV. We used short-term cellular starvation as a strategy to prevent the possibility that the nutrients already present in the culture medium as well as the endogenous substrates would influence the observation of the metabolic alterations induced by infection. This experimental approach was recently developed by our group (26) and allowed us to evaluate the contribution of fatty acids, glutamine, glucose, and pyruvate to mitochondrial oxygen consumption during DENV infection. Interestingly, we found that DENV infection inhibited the Crabtree effect, resulting in an increase in the cellular capacity of metabolizing glucose. Additionally, we further explored the role of glucose in infected cell metabolism, and the results supported the hypothesis that glucose plays an anaplerotic role in the use of endogenous fatty acids. We also found that fatty acids are the main cellular energy source during infection and that pharmacological modulation of β-oxidation has a strong impact in DENV replication.

## RESULTS

### Metabolic switches in DENV-infected Huh7 cells.

**FIG 1:**
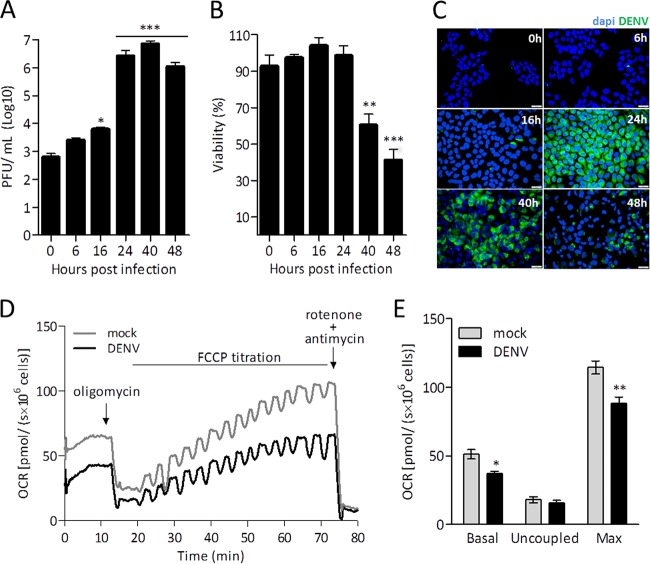
DENV infection inhibits mitochondrial respiration in intact Huh7 cells. HuH7 cells were subjected to mock infection or DENV infection (MOI = 1). (A to C) During the time course of infection, the production of virus infectious particles was quantified by plaque assay (A); cell viability was evaluated by MTT reduction assay (B); and the synthesis of viral proteins was analyzed by immunofluorescence microscopy, using an anti-DENV E protein antibody (green fluorescence) and the nucleus-staining probe DAPI (blue fluorescence) (C). The oxygen consumption rate (OCR) was measured in a suspension of intact cells (2 × 10^6^ cells) in complete DMEM (containing 5 mM glucose, 1 mM pyruvate, and 2.5 mM glutamine), without FBS. (D) Representative results from respirometry experiment performed using mock-infected (gray) and DENV-infected (black) cells. After OCR stabilization, the following ETS modulators were added: oligomycin (0.25 µM) (to measure uncoupled respiration); FCCP (sequential additions to achieve maximum respiration); and rotenone (0.5 mM) and antimycin (3.6 µM) (to determine residual oxygen consumption rates). (E) OCR for mock-infected (white) and DENV-infected (black) cells under the following conditions: basal respiration (Basal); after inhibition of ATP synthase with oligomycin (Uncoupled); upon titration with FCCP until the maximum respiration was reached (Max). Data are represented as means ± standard errors of the means (SEM) of results from 3 independent experiments. *, *P* ≤ 0.05; **, *P* ≤ 0.01 (for comparisons between mock- and DENV-infected cells). In panel C, the magnification used was ×40 and the scale bar corresponds to 10 μm.

A time course of DENV replication in Huh7 cells was evaluated by quantifying the release of infectious particles in the conditioned culture medium and the level of expression of DENV E protein in infected cells (Fig. 1A and B). The virus titer in the conditioned medium increased 2 orders of magnitude after 24 h of infection (Fig. 1A), a time point at which no change in cellular viability was observed (Fig. 1B). Immunofluorescence experiments analyzing E protein also reached the maximum staining at 24 h of infection (Fig. 1C), so all subsequent assays were performed at that time point. High-resolution respirometry was used to evaluate the metabolic shifts in Huh7 cells infected with DENV. Figure 1D shows typical curves of the oxygen consumption rates (OCR) corresponding to infected and mock-infected Huh7 cells incubated in complete culture medium, which contained as the main nutrients 5 mM glucose, 1 mM pyruvate, and 2.5 mM glutamine. The experiment started with the addition of the cells. When the oxygen consumption reached a stable rate, which corresponds to the basal rate of cellular respiration, the following modulators of the respiratory system were added: oligomycin, an inhibitor of ATP synthase; carbonyl cyanide p-(trifluoromethoxy) phenylhydrazine (FCCP), an ionophore that disrupts the proton gradient; and rotenone and antimycin, inhibitors of complexes I and III, respectively. In the presence of oligomycin, protons cannot return to the mitochondrial matrix through ATP synthase, so the measured OCR reflects the proton leakage through the mitochondrial inner membrane and corresponds to the respiration uncoupled to ATP synthesis. FCCP forces the mitochondrial complexes to work at their maximal electron transport capacity. Its titration allows one to measure the maximal rate of respiration that can be reached under the studied condition. In the case of DENV- and mock-infected Huh7 cells, maximal respiration was reached with 400 nM FCCP. Rotenone and antimycin impair electron transport through the respiratory chain and reveal the residual oxygen consumption and the nonmitochondrial respiration plus the oxygen consumption in other mitochondrial reactions besides those occurring in the ETS. This value corresponds to about 5% of the total oxygen consumption and did not vary throughout all the experiments (thus, it is omitted from the results reported below). Comparing the respiratory parameters of DENV- and mock-infected cells, we found that infection inhibits mitochondrial respiration (Fig. 1E). This is seen in basal oxygen consumption and becomes more evident in the maximal respiratory rates. No effect of infection on uncoupled respiration was observed.

**FIG 2:**
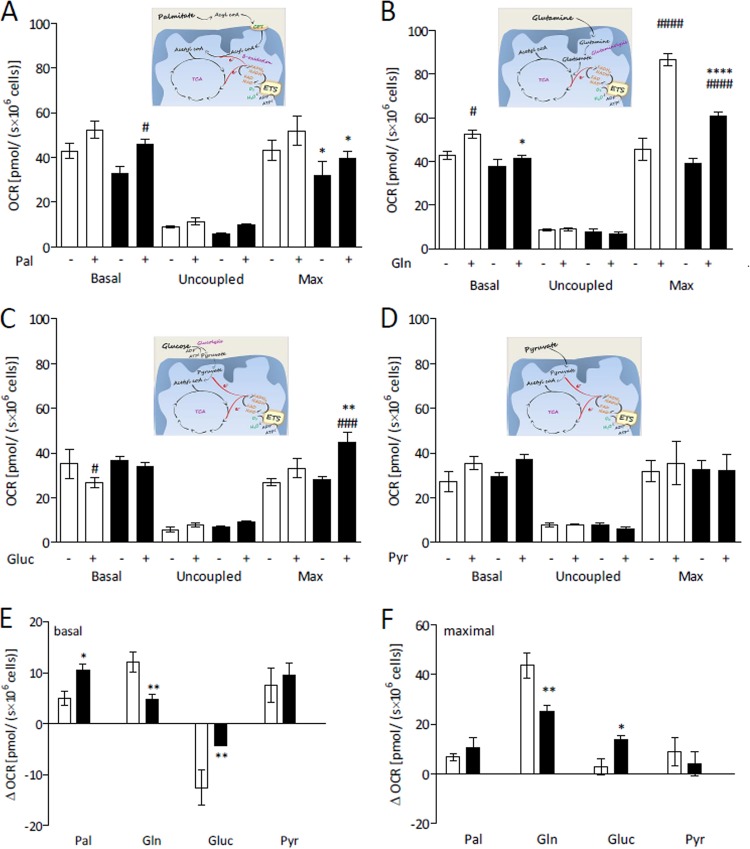
DENV infection alters differentially the use of specific substrates by intact Huh7 cells. HuH7 cells were subjected to mock infection (white) or DENV infection (black) and cultivated in complete DMEM for 24 h. The medium was then replaced by restricted medium (RM; DMEM without glucose, glutamine, and sodium pyruvate) without FBS. After 1 h, the cells were harvested and resuspended in KHB, and the OCR was recorded after addition or not of the following exogenous substrates: (A) 200 µM palmitate (Pal); (B) 2.5 mM glutamine (Gln); (C) 2.5 mM glucose (Glu); or (D) 5 mM pyruvate (Pyr). The following conditions were used: basal respiration; after inhibition of ATP synthase with oligomycin (uncoupled respiration); upon titration with FCCP (maximum respiration [Max]). The schematic figures in each panel illustrate how the respective substrates were oxidized, leading to oxygen consumption. (E and F) Differences between OCR in the presence and in the absence of each substrate (ΔOCR) for coupled respiration values (basal respiration minus oligomycin-insensitive respiration) and maximal respiration values, respectively. Data are represented as means ± SEM of results from at least 4 independent experiments. The asterisks indicate significant differences between mock-infected cells and DENV-infected cells as follows: *, *P* ≤ 0.05; **, *P* ≤ 0.01; ****, *P* ≤ 0.0001. The hash signs indicate significant differences between the results seen in the absence and the presence of the exogenous substrate as follows: #, *P* < 0.05; ###, *P* < 0.001; ####, *P* < 0.0001.

In order to evaluate the use of different substrates by cells, we submitted the cells to a short-term nutrient deprivation protocol, which consisted of cell incubation for 1 h in restricted medium (RM; DMEM [Dulbecco’s modified Eagle’s medium] without glucose, glutamine, and sodium pyruvate) without fetal bovine serum (FBS), before carrying out the experiments. This protocol makes the cells prone to use the chosen substrate without loss of cellular viability, overcoming the influence of the nutrients present in the culture medium (26). Huh7 cells were subjected to mock infection or DENV infection for 24 h, and the OCR was measured when the only substrate added after nutrient deprivation was palmitate, glutamine, glucose, or pyruvate (Fig. 2A to D, respectively). In the figure panels, it is possible to observe separately the cellular responses occurring after addition of each exogenous substrate as well as the effect of infection on the use of these specific substrates. To make more evident the contribution of each oxidative substrate to cellular OCR, differences between the oxygen consumption rates seen in the presence and in the absence of the respective substrates (ΔOCR) under the basal and maximal respiration conditions are shown in Fig. 2E and F, respectively.

The results indicate that infected Huh7 cells effectively use palmitate as an oxidative substrate, since the oxygen consumption in the presence of palmitate was significantly higher than that seen with the control (addition of nonconjugated bovine serum albumin [BSA]) (Fig. 2A). Although the effect of infection on the use of this substrate follows the same profile as that observed in the experiment carried out in complete medium (a tendency toward a decrease in the level of basal respiration that becomes significant for the maximal respiration results; compare Fig. 1B and 2A), the basal ΔOCR data show that infected cells can use palmitate more efficiently than mock-infected cells (Fig. 2E). Glutamine was the most efficacious substrate used by Huh7 cells (Fig. 2B and F). This is very clear when one compares the maximal respiration seen in the absence of any exogenous substrate to that seen in the presence of glutamine, but the data are also significant with respect to the basal rate of respiration of mock-infected cells (Fig. 2B). Remarkably, DENV infection strongly inhibited the use of this nutrient as an energetic substrate for the cells, a much more pronounced effect than that observed in complete medium (compare Fig. 1B and 2B). The infection-induced inhibition of glutamine oxidation was significant in the evaluations of basal respiration (Fig. 2B and E) and became very pronounced for the maximal respiration (Fig. 2B and F). The most striking results were obtained when glucose was used as the exogenous substrate (Fig. 2C, E, and F). Glucose inhibited oxygen consumption by Huh7 cells, as already described for other types of cells (27–29), demonstrating that they undergo the Crabtree effect, clearly indicated by the negative value shown in Fig. 2E. The fact that the basal respiration in mock-infected cells in the presence of glucose was lower than seen in the absence of any exogenous substrate indicates that the decrease of oxygen consumption induced by glucose was related to an inhibition of the use of the endogenous substrates. Surprisingly, DENV infection strongly reduced this effect (Fig. 2E). It is noteworthy that, in the presence of glucose, the maximal oxygen consumption was increased in infected cells, an effect opposite that obtained for the other substrates (Fig. 2F). This explains why the effects of infection observed when glutamine was used as the only substrate were much more evident than those seen in complete medium; the use of glucose by infected cells partially compensated for the infection-induced inhibition of glutamine oxidation. To confirm whether the observed effects were due to glucose and/or the glycolytic activity, we tested the effect of providing pyruvate, the product of glycolysis, to the cells. The results showed that pyruvate, at least at the concentration tested, was not a good oxidative substrate for Huh7 cells and that infection did not affect its use by the cells (Fig. 2D).

### DENV-induced inhibition of the Crabtree effect.

The reduction of the Crabtree effect in DENV-infected Huh7 cells raises two questions. Is glucose oxidation essential during Huh7 infection by DENV? Does glucose become the main energy source for infected cells, being used as an oxidative substrate in mitochondria? The great increase in the maximal level of respiration in the presence of glucose in DENV-infected cells shows that infection enhanced the mitochondrial oxidation of the available substrates, but with this result it was not possible to discriminate whether the exogenous glucose itself or the endogenous stored lipids, for instance (see next topic), represented the main substrate used as an energy source.

**FIG 3:**
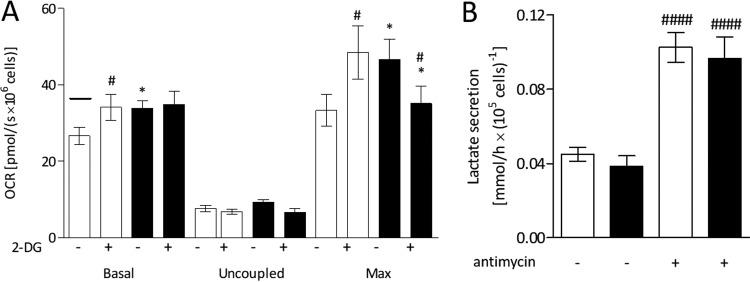
DENV infection increases the level of glucose metabolization but not the cellular glycolytic capacity. HuH7 cells were subjected to mock infection (white) or DENV infection (black) and cultivated in complete DMEM for 24 h. (A) OCR records from intact cells starved for 1 h and then resuspended in KHB supplemented with 5 mM glucose, in the absence or in the presence of 10 mM 2-DG (as indicated in the figure), under the following conditions: basal respiration; after inhibition of ATP synthase with oligomycin (uncoupled respiration); upon titration with FCCP (maximum respiration [Max]). The line over the first bar corresponds to the average OCR seen under the same conditions but in the absence of glucose. (B) Lactate secretion by mock-infected (white) and DENV-infected (black) cells in RM supplemented with 5 mM glucose, in the absence or in the presence of 100 nM antimycin (as indicated in the figure). Data are represented as means ± SEM of results from 4 independent experiments. The asterisks indicate significant differences between mock- and DENV-infected cells as follows: *, *P* ≤ 0.05. The hash signs indicate significant differences between the absence and the presence of the inhibitors as follows: #, *P* ≤ 0.05; ####, *P* ≤ 0.0001.

To answer these questions, we first evaluated the effects of infection on cellular glycolytic activity. We performed respirometry experiments in the presence of 2-deoxyglucose (2-DG), a molecule that impairs the use of glucose through the glycolytic pathway by competing with glucose as the substrate of the first glycolytic enzyme, hexokinase. This experiment resulted in a number of interesting observations (Fig. 3A). In the presence of 2-DG, the OCR recovered to values similar to those observed in the absence of glucose (indicated by the line in Fig. 3A), demonstrating that 2-DG abolished the Crabtree effect. For the infected cells, 2-DG had no effect on the basal level of respiration. This can be explained by the fact that the Crabtree effect did not occur in infected cells, as already shown in Fig. 2C. Intriguingly, the maximal rate of respiration was significantly increased in the presence of 2-DG. A possible explanation for this result would be that 2-DG favored oxidative phosphorylation by increasing ADP availability, since its phosphorylation by hexokinase converted ATP in ADP, which was not reconverted into ATP in the glycolysis reactions that followed. Finally, 2-DG abolished the increase in the maximal oxygen consumption observed for the infected cells in the presence of glucose, supporting the hypothesis that DENV infection increases the level of glucose metabolization. However, comparing the levels of lactate production between infected and mock-infected cells, we found that the glycolytic flow was not altered by infection; the levels of lactate production were the same in DENV- and mock-infected cells, even when oxidative phosphorylation was inhibited by antimycin (Fig. 3B), indicating that DENV infection does not lead to activation of fermentative metabolism. Thus, the role of glucose in the stimulation of the oxidative metabolism observed in infected cells (increase in maximal respiration) cannot be explained by an increase in the glycolytic capacity, suggesting that glucose utilization in infected cells would favor the oxidization of endogenous substrates.

### Fatty acid oxidation as the main energy source in DENV-infected cells.

**FIG 4:**
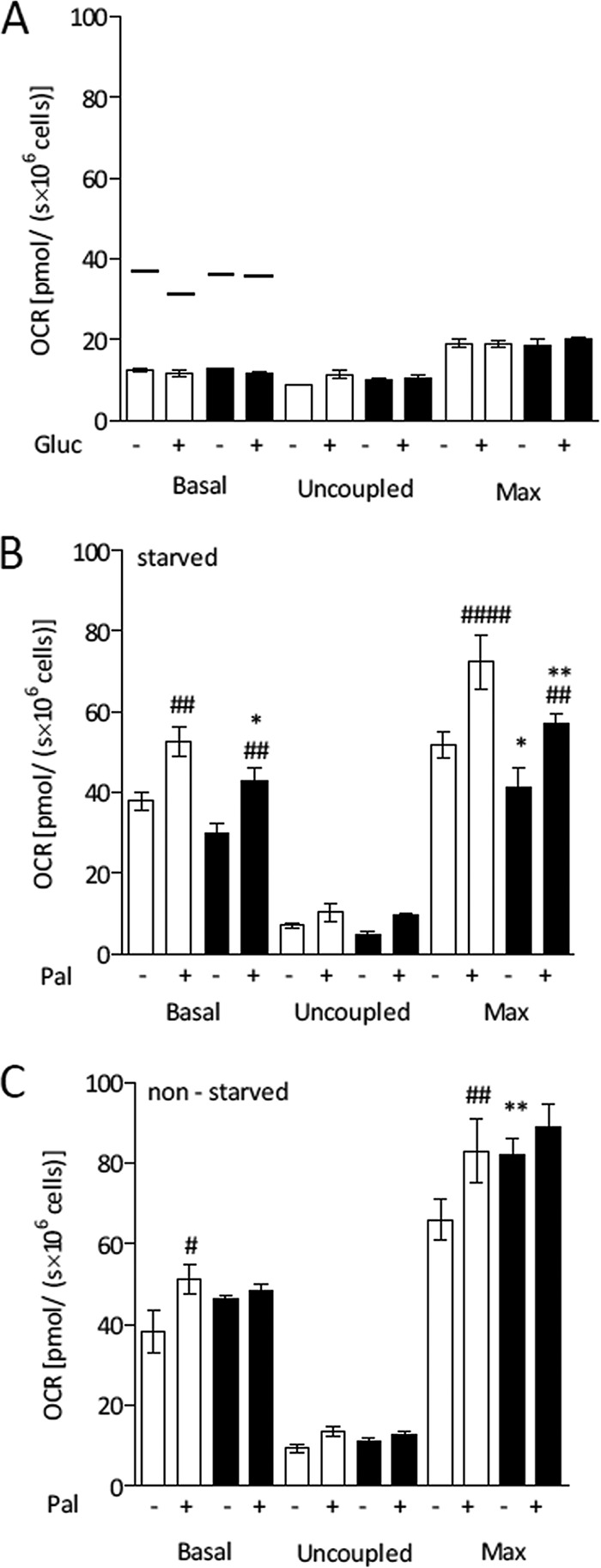
Glucose metabolization increases endogenous oxidation of fatty acids in DENV-infected Huh7 cells. HuH7 cells were subjected to mock infection (white) or DENV infection (black) and cultivated in complete DMEM for 24 h. OCR measurements were performed under the following conditions: basal respiration; after inhibition of ATP synthase with oligomycin (uncoupled respiration); upon titration with FCCP (maximum respiration [Max]). For the experiments represented in panels A and B, the cells were maintained for 1 h in restricted medium (RM) without FBS before the experiment (starved cells). For the experiment represented in panel C, that step was omitted (nonstarved cells). (A) OCR records of intact cells in KHB in the presence of 200 μM etomoxir, after addition or not of 5 mM glucose (Gluc [as indicated in the figure]). The lines over the bars correspond to the average OCR levels seen under the same conditions but in the absence of etomoxir. (B and C) OCR records of intact cells in KHB in the presence of 5 mM glucose, after addition or not of 200 µM palmitate (Pal, as indicated in the figure). Data are represented as means ± SEM of results from at least 4 independent experiments. The asterisks indicate significant differences between mock- and DENV-infected cells as follows: *, *P* ≤ 0.05; **, *P* ≤ 0.01. The hash signs indicate significant differences between the results seen in the absence and the presence of the exogenous substrate as follows: #, *P* ≤ 0.05; ##, *P* ≤ 0.01; ####, *P* ≤ 0.0001.

Intense fatty acid oxidation requires an increased supply of Krebs cycle intermediates in order to complete the oxidation of the high number of acetyl-CoA (acetyl-CoA) molecules produced in β-oxidation. This is usually provided by the conversion of pyruvate to oxaloacetate, catalyzed by the mitochondrial enzyme pyruvate carboxylase. Thus, if fatty acid β-oxidation is the main source of energy in infected cells, the increasing rates of pyruvate formation in glycolysis would have this anaplerotic role. Therefore, to evaluate whether the glucose metabolization in DENV-infected cells favors fatty acid oxidation, we analyzed oxygen consumption in the presence of glucose after adding etomoxir, an inhibitor of carnitine-palmitoyl transferase-1 (CPT-1), an enzyme involved in the transport of the acyl-CoA molecules to the mitochondrial matrix, where they undergo β-oxidation (Fig. 4A). The results showed that oxygen consumption in the presence of etomoxir decreases to levels almost as low as those corresponding to the proton leak, despite the presence or absence of glucose, sustaining the hypothesis that oxygen consumption in the presence of glucose is mostly associated with the use of endogenous fatty acids and not with an oxidative use of the glucose itself. It is important that the specificity of etomoxir in inhibiting fatty acid oxidation, while not affecting the use of the other substrates, was confirmed here by measuring the OCR of etomoxir-treated cells after addition of exogenous glutamine, as well as that of cells oxidizing glutamine after etomoxir addition (not shown).

To further investigate the dependence on glucose for fatty acid oxidation in Huh7 cells, we evaluated oxygen consumption after adding exogenous palmitate in the presence of glucose (Fig. 4B). The results showed that glucose did facilitate the cellular capacity of oxidizing exogenous palmitate. If one compares the results of the experiments performed in the absence and in the presence of glucose (Fig. 2A and 4B, respectively), this is evident for the basal oxygen consumption but becomes even clearer for the maximal respiration. To evaluate whether a higher level of availability of fatty acids would increase the cellular respiratory capacity, we performed the same experiment using nonstarved cells (i.e., cells not submitted to the nutrient deprivation protocol), which have the energy stores preserved (Fig. 4C). It is noteworthy that under this condition, both the basal and maximal rates of respiration were higher in infected cells, even in the absence of exogenous palmitate, confirming that glucose utilization in DENV-infected cells increases the oxidation capacity of endogenous fatty acids. In agreement, the levels of glucose-stimulated oxidation of palmitate in starved and nonstarved mock-infected cells were quite similar (compare Fig. 4B and C).

### DENV replication depends on fatty acid oxidation.

**FIG 5:**
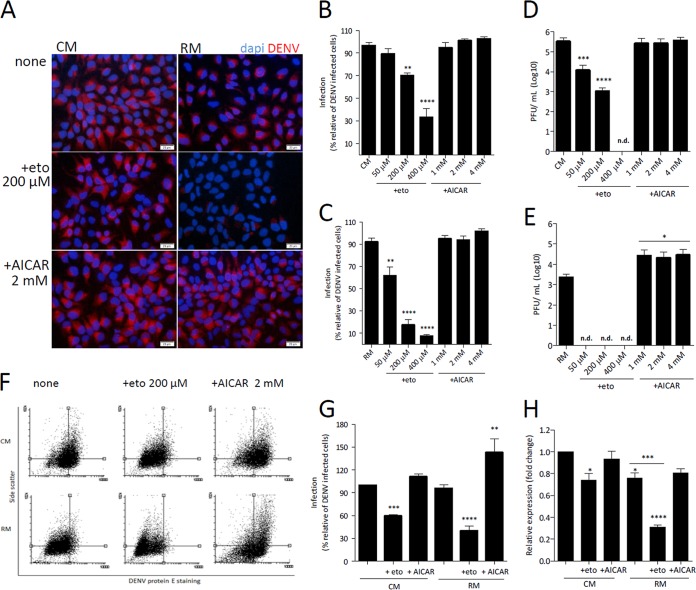
DENV replication depends on fatty acid β-oxidation. HuH7 cells were infected with DENV (MOI = 1) and cultivated in complete DMEM for 20 h. The medium was then replaced by complete medium (CM) or restricted medium (RM) containing 50, 200, or 400 μM etomoxir or 1, 2, or 4 mM AICAR, as indicated in the figure, and the cells were cultivated for an additional 4 h, completing 24 h of infection. The conditioned media were collected for quantification of infectious virus particles, and cells were processed for immunofluorescence microscopy or flow cytometry, using anti-DENV E protein polyclonal antibody. (A) Representative immunofluorescence images of infected cells stained for DENV E protein (red fluorescence) after incubation with 200 µM etomoxir (eto) or 2 mM AICAR in complete medium (CM) or restricted medium (RM). Cell nuclei were stained with DAPI (blue fluorescence). Magnification, ×20; bar, 20 μm. (B and C) Quantification of DENV-positive cells in the immunofluorescence images of cells treated with 50, 200, or 400 μM etomoxir or with 1, 2, and 4 mM AICAR in CM (B) or RM (C). The analyses were performed using ImageJ software. (D and E) Quantification of DENV infectious particles in the conditioned medium of cells treated with 50, 200, or 400 μM etomoxir or with 1, 2, and 4 mM AICAR in CM (D) or RM (E). n.d., not determined. (F) Representative dot plots showing the cellular levels of DENV E protein staining versus cellular side scatter. As references for better visualization of cell distribution, quadrants were fixed in the center of the data corresponding to the nontreated infected cell populations for each condition (CM or RM). (G) Quantification of DENV-positive cells after treatment with 200 μM etomoxir or 2 mM AICAR in CM or RM using CellQuest software. (H) DENV RNA replication measured by real-time PCR. Data are represented as means ± SEM of results from 3 independent experiments. The asterisks indicate significant differences between treated and untreated cells: *, *P* ≤ 0.05; **, *P* ≤ 0.01; ***, *P* ≤ 0.001; ****, *P* ≤ 0.0001. The hash signs indicate significant differences between the complete medium and restricted medium as follows: #, *P* ≤ 0.01.

Finally, we analyzed whether the modulation of β-oxidation affected DENV replication in Huh7 cells. For this, we evaluated DENV replication after pharmacologically inhibiting or stimulating fatty acid β-oxidation using etomoxir or AICAR (5-aminoimidazole-4-carboxamide ribonucleotide), respectively (Fig. 5). The experiments were carried out using cells maintained in complete culture medium or submitted to nutrient deprivation. Cell treatment with etomoxir significantly inhibited in a dose-dependent manner the expression of DENV E protein, measured by fluorescence microscopy (Fig. 5A to C), as well as the formation of infectious viral particles, measured by plaque assay (Fig. 5D and E), showing the requirement of β-oxidation for a productive DENV infection. The effects of etomoxir treatment were more pronounced when the cells were maintained in medium without the main nutrients. In the case of treatment with AICAR, the opposite was found. Although fluorescence microscopy analyses did not show an increase in the number of DENV-positive cells (Fig. 5A to C), quantification of E protein-positive cells and of cellular internal complexity by flow cytometry (Fig. 5F and G), as well as of the formation of infectious viral particles, measured by plaque assay (Fig. 5D and E), showed that treatment with AICAR enhanced DENV infection. The dependence on fatty acid oxidation for DENV replication was also confirmed by real-time PCR (Fig. 5H). It is important that neither etomoxir nor AICAR had any effect on cell viability under all the conditions tested (data not shown).

## DISCUSSION

Depending on the metabolic status and nutrient availability, mitochondrial oxidative machinery switches among the main energetic substrates—fatty acids, glucose, and amino acids. However, under some pathological conditions, this metabolic flexibility may be impaired, leading to cellular dysfunction and/or damage to the organism. Here we present a detailed analysis of the use of different exogenously added substrates by Huh7 cells, showing strong differences among the substrates evaluated and revealing that DENV infection affects the use of each substrate in distinct manners. In summary, the results showed that whereas infection strongly inhibits glutamine oxidation, it favors the cellular capacity of metabolizing glucose, allowing its anaplerotic role in the use of endogenous fatty acids. We found that these molecules become the main energetic substrate during infection, being essential for virus replication. Another remarkable finding was that DENV infection inhibits that Crabtree effect, and the results, taken together, allowed us to propose a mechanism to explain this intriguing phenomenon in the context of infection.

Our results clearly show that Huh7 cells can use palmitate efficiently as a respiratory substrate. Unfortunately, due to an experimental constraint, it is not possible to compare quantitatively the oxygen consumption values obtained for palmitate with those observed for the other substrates, since fatty acids are known for their uncoupling effect on mitochondrial oxidative phosphorylation (30, 31). Here we found that the maximal palmitate concentration that allowed reliable measurement of oxygen consumption coupled to ATP synthesis was 200 µM, much less than the concentrations used for glutamine, glucose, or pyruvate (used in the millimolar range). A previous study aimed at evaluating the role of mitochondrial metabolism in hepatocyte lipotoxicity showed an increase in oxygen consumption after cell incubation with 400 µM palmitate (32). Those researchers did not use oligomycin to quantify palmitate-induced uncoupling; however, the fact that they showed that palmitate-stimulated oxygen consumption remained as high as in the control cells after the addition of a complex I inhibitor indicates that it corresponds entirely to an uncoupling effect of the fatty acid rather than to the palmitate oxidation itself. In the case of the experiments shown here, using a lower palmitate concentration, we not only could measure the coupled respiration associated with palmitate oxidation but also found that it reaches the same levels as those obtained for glutamine or pyruvate.

**FIG 6:**
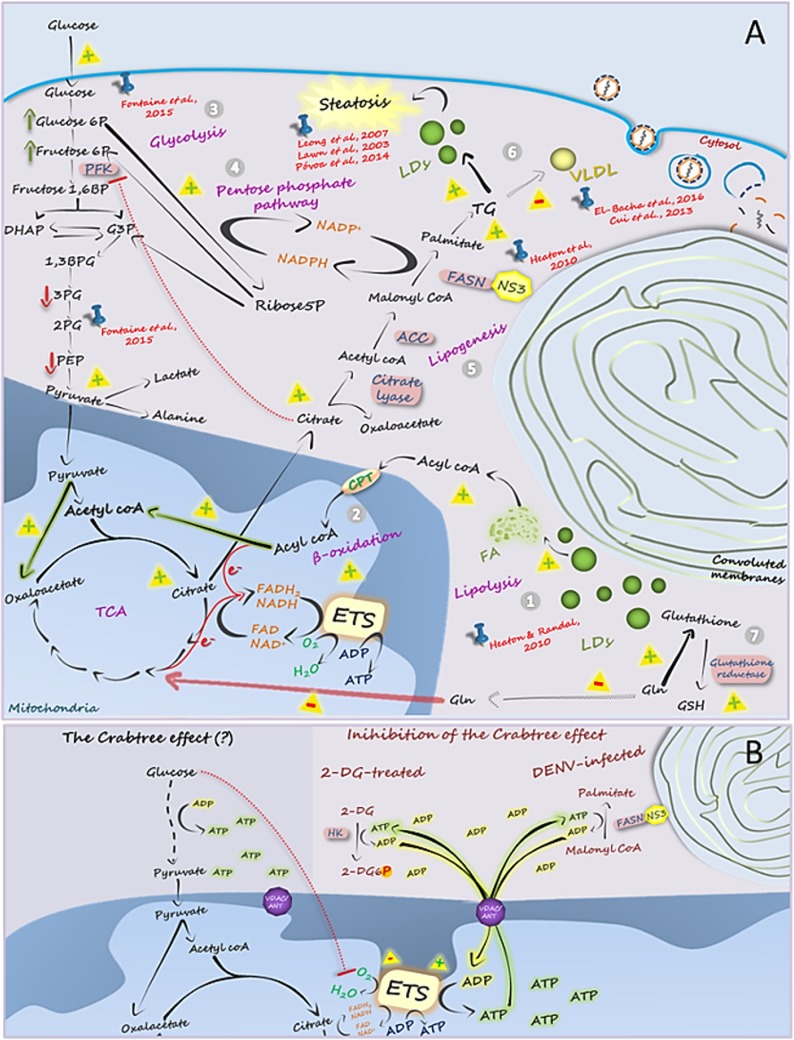
Integrative view of DENV-induced metabolic alterations in hepatic cells. (A) (Step 1) Mobilization of fatty acids from LDs appears to be essential for DENV replication, as supported by the results obtained by Heaton and Randall (6). Fatty acids are transported to the mitochondrial matrix, where β-oxidation works as the main energy-supplying pathway for maintaining virus replication, as we show here. (Step 2) The oxidation of the large contingent of acetyl-CoA molecules generated in β-oxidation requires tricarboxylic acid (TCA) cycle feeding with oxaloacetate. Our results support the idea of an anaplerotic role of glucose in this context, with the pyruvate molecules generated in glycolysis being converted into oxaloacetate by the pyruvate carboxylase (PC) reaction. (Step 3) Indeed, here we have shown that glucose has great importance in the oxidation of endogenous fatty acids in DENV-infected cells, which may explain the high glucose uptake and the increase in its requirement during DENV replication, as reported by Fontaine et al. (20). Interestingly, those authors showed in the same work an increase in the initial intermediates of glycolysis, glucose-6-phosphate, and fructose-6-phosphate and a reduction in the final intermediates of this pathway, 3-phosphoglycerate, phosphoenolpyruvate, and lactate. (Step 4) Although Fontaine et al. (20) interpreted their observations to represent the result of an activation of the glycolytic pathway during infection, we propose here an alternative view for these findings: infection would activate the pentose-phosphate pathway, which not only diverts the available glycolysis intermediates to use in other reactions but also provides NADPH for the fatty acid synthesis reactions. Accordingly, glucose may also be a source of carbons in the formation of ribose-5-phosphate, an important intermediate for the synthesis of nucleic acids which is required during viral replication. (Step 5) Heaton et al. (22) showed that DENV NS3 recruits fatty acid synthase (FASN) to the viral replication sites, stimulating its activity and, consequently, the synthesis of fatty acids in DENV-infected hepatic cells. Fatty acid synthesis is triggered by the accumulation of citrate in the cytosol, which acts both as the carbon source for malonyl-CoA formation and as a positive regulator of acetyl-CoA carboxylase (ACC). In addition, citrate is an important negative regulator of the enzyme phosphofructokinase (PFK), causing the accumulation of the initial glycolysis intermediates (as observed by Fontaine et al. [20]) and favoring the glucose-6-phosphate shift toward the pentose-phosphate pathway, which generates, besides ribose-5-phosphate, NADPH, the reduced coenzyme required by FASN reactions. (Step 6) Fatty acids are required for the synthesis of either the convoluted membranes or triacylglycerols (TGs), which might be stored in LDs, culminating with hepatic steatosis, already observed in DENV infection by Leong et al. (15), Lawn et al. (47), and Póvoa et al. (16). The accumulation of lipids in the intracellular LDs seems to reduce the production of very-low-density lipoproteins (VLDL), as already shown in the clinical studies by Cui et al. (10) and El-Bacha et al. (14). (Step 7) Finally, here we showed that infection reduces the ability of hepatic cells to oxidize glutamine. This would be explained by the fact that this amino acid is essential for formation of glutathione, which in the reduced form (GSH) is an important antioxidant molecule. Glutamine may also be converted into other amino acids required during viral replication. The signals “+” and “-” indicate the steps that are activated and inhibited, respectively, during DENV infection, based on the literature (cited in red in the figure) or on the results presented here (highlighted by the arrows colored in green, in the case of the steps that we showed to be activated, or in red, in the case of the steps that we showed to be inhibited). (B) Proposed mechanism for the Crabtree effect. Here we found that both DENV infection and 2-DG treatment reverse the Crabtree effect in hepatic cells. Since in both cases cytoplasmic ATP-consuming reactions are stimulated (fatty acid synthesis in infection and phosphorylation of 2-DG by hexokinase after cellular treatment with this molecule), our results support the most accepted hypothesis to explain the Crabtree effect: the intense ATP synthesis during glycolysis results in a decrease in ADP availability, reducing its uptake by mitochondria, which impairs oxidative phosphorylation.

Although our results show that Huh7 cells possess a high capacity of oxidizing glutamine, DENV infection strongly inhibited the use of this amino acid as an energy source for the cells. One explanation for this observation would be that, instead of being used as an energetic substrate, glutamine is used for other metabolic fates required during infection, as in the case of supplying amine groups for nucleotide and UDP *N*-acetylglucosamine biosynthesis, or as a source of glutamate for glutathione synthesis (33), which is important to protect the cells against infection-induced oxidative stress (Fig. 6A). Glutamate may also be converted to another amino acid by transamination, sustaining the intense viral protein synthesis that occurs during infection. The fact that the plasma levels of glutamine were found to be decreased in dengue patients (10, 14) supports the hypothesis that, rather than being unnecessary for DENV replication, the role of glutamine is shifted from that of an energetic source to that of a biosynthetic precursor in infected cells. In contrast, Fontaine and coworkers (20) showed that depriving human foreskin fibroblasts (HFF) of exogenous glutamine did not have an impact on DENV replication, a result that would be explained by the rates of DENV replication in HHF being lower than the rates seen in hepatic cells.

The analysis of glucose oxidation by DENV-infected Huh7 cells opened a series of very interesting and unexplored lines of investigation. The first surprising result was the inhibition of the Crabtree effect by infection. The Crabtree effect has been described in different types of cells, including hepatocytes (34), but, to our knowledge, virus-induced inhibition of this effect has never been reported so far. At first glance, this result suggests that glucose would become an important energy source for DENV-infected hepatocytes, as also proposed for infected primary human fibroblasts (20). However, by measuring lactate secretion levels, we found no changes in the glycolytic flow of infected cells, suggesting that the role of glucose in infected cell metabolism is more complex, supporting an interpretation different from that reported for the previous study. There, metabolomics analyses revealed that the concentrations of early glycolytic metabolites (glucose-6-phosphate and fructose-6-phosphate) increased with DENV infection whereas the concentrations of late glycolytic metabolites (3-phosphoglycerate and phosphoenolpyruvate) decreased (20). These results occurred together with an increase in the expression of GLUT-1 (and an increase in glucose uptake) and hexokinase genes. Although the authors concluded that infection activates the glycolytic pathway, these data would be interpreted as being a consequence of the activation of pentose phosphate and fatty acid synthesis pathways, both of which are processes are required during infection as they provide lipids and nucleotides for viral replication. During fatty acid synthesis, the citrate concentration in the cytosol increases, inhibiting phosphofructokinase 1 (PFK-1) and leading to an accumulation of fructose-6-phosphate and glucose-6-phosphate, the latter being shunted to the pentose-phosphate pathway (Fig. 6A). This pathway provides not only ribose-5-phosphate for nucleotide synthesis but also NADPH, which is required for fatty acid synthase (FASN) activity. In fact, during DENV infection, FASN is recruited to the replication complexes, where FASN-NS3 interaction results in the activation of fatty acid synthesis in the sites of viral replication (22). Taken together, these results support the hypothesis that the role of glycolysis during DENV infection is to act as the carbon source for fatty acid synthesis as well as to feed the pentose-phosphate pathway to generate NADPH, which represents the reductive power required for this process. It is interesting that fatty acid synthesis is an ATP-consuming pathway, which would result in a great increase in the availability of cytoplasmic ADP in infected cells. This is in agreement with the infection-induced inhibition of the Crabtree effect described here (Fig. 6B), as the most accepted hypothesis to explain this phenomenon is based on the lack of ADP availability due to the formation of large amounts of cytoplasmic ATP during glycolysis, thus decreasing ADP uptake by mitochondria and reducing the capacity of the ETS to synthesize ATP (35). Accordingly, the result obtained here for 2-DG-treated cells also supports this hypothesis. The reduction of the Crabtree effect observed for 2-DG-treated cells may be explained by the fact that 2-DG is phosphorylated by hexokinase at the expense of ATP, thus also increasing ADP availability. The increase in the ADP levels either in infected cells or in 2-DG-treated cells would favor oxidative phosphorylation. Thus, the inhibition of the Crabtree effect both in DENV-infected cells and in 2-DG-treated cells supports the idea that it is related to the increase in ADP availability in both situations, reinforcing the likelihood that it is the mechanism behind this intriguing metabolic phenomenon, which is still not well understood (Fig. 6B).

Another important contribution of our work was to shed light on the identity of the main substrate feeding oxidative phosphorylation during DENV infection. Although an increased capacity of using glucose was clearly observed for the infected cells, the experiment carried out in the presence of etomoxir revealed that oxygen consumption in the presence of glucose is mainly associated with the β-oxidation of endogenous fatty acids. Indeed, Heaton and Randall showed that DENV replication in Huh7.5 cells (a subline derived from Huh7 cell line) activates β-oxidation, which is fed by mobilization of triacylglycerols from LDs (6) (Fig. 6A). Additionally, glucose also facilitates the cellular capacity of oxidizing exogenous fatty acids. Thus, taken together, the results show that glucose utilization in DENV-infected cells increases the cellular capacity of oxidizing endogenous or exogenous fatty acids and that β-oxidation is the main ATP-generating pathway necessary to maintain the cellular functions and the high energy demands of viral replication (Fig. 6A).

Interestingly, although the fatty acid synthesis and β-oxidation pathways are expected to be antagonistically regulated, it is likely that the two processes occur simultaneously, and at high rates, in DENV-infected cells. At the first glance, this seems contradictory, but the differences in the subcellular localizations of the two processes as well as the huge cellular alterations caused by infection, especially the formation of the membranous replication complexes, would explain this unusual metabolic behavior: while lipid biosynthesis occurs at viral replication sites, providing the precursors for membrane synthesis, fatty acids seems to be mobilized from LD to be oxidized in mitochondria, providing energy for virus replication. This may also explain the two antagonistic observations also related to lipid metabolism in DENV-infected hepatic cells. While Jordan and Randall showed that infection leads to the activation of the AMP-activated protein kinase (AMPK), inducing lipophagy and thus lipid oxidation (36), Soto-Acosta et al. reported that upregulation of cholesterol synthesis in DENV-infected cells occurs due to AMPK inhibition during infection (37). It is possible that these apparently controversial observations resulted from very different regulation processes occurring in distinct subcellular compartments.

The idea of the role of β-oxidation as the main energy-yielding pathway during DENV infection is also supported by the increased viral replication levels observed for AICAR-treated cells. AICAR is an AMP analog that stimulates AMPK, which, in turn, phosphorylates acetyl-CoA carboxylase (ACC), inhibiting its activity with respect to synthesizing malonyl-CoA. Since malonyl-CoA inhibits CPT-1 (as etomoxir does also), impairing the transport of fatty acids into the mitochondria, the increase in DENV replication in the presence of AICAR corroborates the role of fatty acid β-oxidation in sustaining DENV infection and is in agreement with the previous report showing the activation of AMPK in infected cells (36). The fact that an AICAR analog does not impact DENV NS3 activity (38) suggests that the increase in AICAR-induced DENV replication is not a direct effect on viral proteins but a metabolic driven process.

Recently, it was demonstrated that DENV infection induces mitochondria elongation and that this event is essential for DENV replication (39, 40). As elongated mitochondria (originating from mitochondria fusion) are known to work more efficiently and are associated with a more oxidative type of behavior (41), those studies support our findings that DENV-infected cells are more dependent on mitochondrial oxidative metabolism for supporting their bioenergetics requirements and that fatty acids are essential substrates in this context.

## MATERIALS AND METHODS

### Cell culture and virus infection.

DENV serotype 2, strain 16681, was propagated in theC6/36 *Aedes albopictus* cell line, as described elsewhere (42). Virus titers were determined by plaque assay (43). Huh7 cells, a human hepatocarcinoma cell line, were cultured in DMEM with 5 mM glucose (Gibco, USA), supplemented with 10% fetal bovine serum (FBS) (Invitrogen, USA), 100 U/ml penicillin, 100 g/ml streptomycin, 0.22% sodium bicarbonate, and 0.2% HEPES (pH 7.4), in a CO_2_ humid incubation chamber, at 37°C. Cells were seeded on 60-cm^2^ plastic petri dishes at a density of 10^5^ cells per ml. After 24 h, cells were subjected to either mock infection or DENV infection using a multiplicity of infection (MOI) of 1 PFU per cell. The assays were carried out 24 h after infection. In the experiments in which the effects of modulation of β-oxidation on DENV replication were tested, the cells were washed with phosphate-buffered saline (PBS) 20 h after infection, and etomoxir (Sigma-Aldrich, St. Louis, MO, USA) (50, 200, or 400 µM, as indicated in the figures), a carnitine-acyl transferase I (CPT-1) inhibitor, or AICAR (AICAR) (Sigma-Aldrich) (1, 2, or 4 mM, as indicated in the figures), an activator of AMP-activated protein kinase (AMPK), was added to the cultures in fresh DMEM with 5 mM glucose (complete medium [CM]) or in DMEM without glucose, glutamine, or pyruvate (restricted medium [RM]). The cells were then maintained for 4 more hours, and the assays were performed. This protocol was used to allow β-oxidation to be modulated at the peak of viral replication (24 h postinfection) but while avoiding the toxic effects of the compounds (cells treated for longer periods showed that their viability was affected). Intracellular viral protein E was quantified by immunofluorescence and flow cytometry. Cell viability was verified using a 3-(4,5-dimethylthiazol-2-yl)-2,5-diphenyl tetrazolium bromide (MTT) (USB, Ohio, USA) assay and through membrane integrity analysis using a Muse count and viability kit (Merck Millipore, Darmstadt, Germany), following the manufacturer’s instructions.

### Immunofluorescence.

Huh7 cells were grown on glass coverslips in 24-well plates, being subjected to mock infection or DENV infection (MOI = 1) after reaching 80% confluence. DENV replication was determined by analyzing the levels of expression of DENV E protein in infected cells by immunofluorescence. To evaluate the time course of virus replication, the cells were fixed with 3.7% formaldehyde solution for 10 min, at room temperature, at different time points after infection. To determine the effects of the modulation of fatty acid oxidation on DENV replication, the cells were treated with etomoxir or AICAR at the concentrations indicated in the figures, 20 h postinfection, and fixed with 3.7% formaldehyde 4 h after the treatments. Fixed cells were washed with PBS, permeabilized with 0.1% Triton X-100 for 4 min, and incubated with PBS supplemented with 1% BSA for 30 min to block nonspecific antibody binding sites. The cells were then incubated with anti-DENV E protein antibody MAB8705 (Chemicon, Merck Millipore) at a 1:600 dilution, for 1 h, and subsequently with goat anti-mouse IgG Cy5.5-conjugated antibody (Jackson Immuno Research) at a 1:800 dilution or Alexa Fluor 488 (Abcam, Inc., MA) at 1:600, for 1 h. Stained coverslips were mounted with Prolong Gold with DAPI (4′,6-diamidino-2-phenylindole) (Invitrogen), and images were acquired using an Olympus IX81 microscope and a 20× or 40× objective. Images were captured using CellSens Standard software and processed with ImageJ software (National Institutes of Health).

### Oxygen consumption analyses.

Mitochondrial oxygen consumption rates (OCR) were measured by high-resolution respirometry using an Oxygraph-2k instrument (Oroboros Instruments, Austria). OCR were measured in intact Huh7 cells suspended in Krebs-Henseleit buffer (KHB), which contains 1.1 M NaCl, 47 mM KCl, 20 mM MgSO_4_, 12 mM Na_2_HPO_4_, and 5.5 mM HEPES. The cells were washed with PBS and removed from the plates by incubation with a 0.25% (wt/vol) EDTA-trypsin solution. The cell suspension was centrifuged for 5 min at 1,200 rpm, and the pellet was resuspended in KHB to approximately 2 × 10^6^ cells in the 2-ml oxygraph chamber. During the experiments, the cells were maintained in suspension by stirring at a rate of 750 rpm. For the experiments in which the oxidation of specific substrates was evaluated, the cells used had been previously submitted to a short-term starvation protocol: the cells were washed with phosphate-buffered saline (PBS) and incubated for 1 h in RM, supplemented with 0.22% sodium bicarbonate and 0.2% HEPES. For measuring fatty acid oxidation, RM was supplemented with 0.5 mM carnitine and 200 μM palmitate conjugated to acid-free bovine serum albumin (BSA), following the protocol developed by Seahorse Bioscience Inc. (North Billerica, MA); the control condition for these assays consisted of a preincubation with BSA alone. After the cells were added to the oxygraph chamber, different respiratory parameters were assessed. Basal OCR were recorded after signal stabilization. Addition of oligomycin (0.25 nM final concentration) allows the evaluation of oxygen consumption uncoupled to the ATP synthesis, referred here as uncoupled respiration. Oxygen consumption coupled to the ATP synthesis (coupled respiration) is calculated by subtracting the value corresponding to uncoupled respiration from the basal OCR value. The maximum OCR was measured in the presence of the optimum concentration of carbonyl cyanide p-(trifluoromethoxy) phenylhydrazone (FCCP), determined after FCCP titration (40 to 600 nM). Finally, the residual OCR (Rox) was obtained using 0.5 mM rotenone and 3.6 nM antimycin to inhibit mitochondrial respiratory complexes I and III, respectively. The value corresponding to the residual OCR was around 5% of the basal OCR value for all experiments and was not considered in the analyses. For inhibition of glycolysis or β-oxidation, 10 mM 2-deoxyglucose (2-DG) or 200 μM etomoxir, respectively, was added to the oxygraph chamber at the beginning of the experiments. Data acquisition and analyses were carried out using DatLab 4.3 software (Oroboros Instruments, Innsbruck, Austria).

### Lactate secretion.

Huh7 cells were subjected to mock infection or DENV infection (MOI = 1). After 24 h, the monolayers were washed twice with PBS, at 37°C, and incubated with RM supplemented with 5 mM glucose, in the presence or absence of antimycin (3.6 nM), for 1 h at 37°C in a CO_2_ humid incubation chamber. Then, the conditioned culture medium was collected and lactate levels were quantified by lactate dehydrogenase enzymatic assay in hydrazine/glycine buffer, following spectrophotometric measurement of the formation of NADH, as described previously (44, 45). Data were normalized according to the number of cells.

### Flow cytometry.

Huh7 cells were subjected to mock infection or DENV infection (MOI = 1), and after 20 h, the cells were treated with 200 µM etomoxir or 2 mM AICAR and maintained for 4 more hours. Then, the cells were washed with PBS, harvested, fixed in 4% paraformaldehyde for 15 min, treated with 0.1% saponin–PBS, and incubated with blocking solution (PBS supplemented with 2% FBS and 0.1% BSA) for 30 min, at room temperature. Then, cells were incubated for 1 h with mouse anti-DENV polyclonal antibody MAB8705 (Chemicon, Merck Millipore), washed, and stained with anti-mouse IgG conjugated to Alexa Fluor 488 (Invitrogen, Carlsbad) for 30 min. The percentage of DENV-infected cells was evaluated by using a FACSCalibur cytometer (Becton Dickinson immunocytometry system). For each sample, 10,000 events were acquired and analyzed using CellQuest software.

### Quantitative PCR of DENV genome.

Huh7 cells were infected and treated with etomoxir or AICAR as described for the flow cytometry experiments. Total cellular RNA was extracted with a GE Healthcare illustra RNAspin isolation kit. Reverse transcription was performed using a High-Capacity cDNA reverse transcription kit (Applied Biosystems) according to the instructions of the manufacturer. Quantitative PCR of DENV RNA was performed as described previously (46) using TaqMan Universal PCR master mix (Applied Biosystems). Data are represented with relative quantification values determined using the threshold cycle (2^−Δ*CT*^) formula where Δ_*CT*_ corresponds to *C*_*T*treated_ − *C*_*T*control_.

### Statistical analyses.

Statistical analyses were performed using GraphPad Prism 7.0 (GraphPad Software, Inc.). Results are presented as means ± standard errors (SE) and were compared by two-tailed Student’s *t* test or were analyzed by one-way analysis of variance (ANOVA) and Student-Newman-Keuls multiple-comparison tests. *P* values of ≤0.05 were considered significant.
